# Years of life lost due to premature deaths associated with air pollution: an ecological time-series study

**DOI:** 10.1590/1516-3180.2021.0129.090422021

**Published:** 2021-10-29

**Authors:** Luiz Fernando Costa Nascimento, Luciana Cristina Pompeo Ferreira Vieira

**Affiliations:** I MD, PhD. Researcher, Postgraduate Program on Mechanical Engineering, Department of Energy, Universidade Estadual de São Paulo (UNESP), Guaratinguetá, Brazil.; II MSc. Doctoral Student, Postgraduate Program on Mechanical Engineering, Department of Energy, Universidade Estadual de São Paulo (UNESP), Guaratinguetá, Brazil.

**Keywords:** Mortality, premature, Particulate matter, Air pollutants, Value of life, DALY, Financial costs, Years of life lost

## Abstract

**BACKGROUND:**

Exposure to air pollutants is associated with hospital admissions due to cardiovascular diseases and premature deaths.

**OBJECTIVE:**

To estimate years of life lost (YLL) due to premature deaths and their financial costs.

**DESIGN AND SETTING:**

Ecological time-series study carried out in São José dos Campos, Brazil, in 2016.

**METHODS:**

Data on deaths among residents of this city in 2016 were assessed to estimate the financial cost of premature deaths associated with air pollution. The diagnoses studied were ischemic heart disease, congestive heart failure and cerebrovascular disease, according to YLL. The fractions attributable to deaths associated with air pollutant exposure and to each potential year of life lost were calculated using negative binomial regression with lags of 0-7 days between exposure and outcome. Nitrogen dioxide, particulate matter (PM_10_) and ozone concentrations were included in the model and adjusted for temperature, humidity and seasonality.

**RESULTS:**

Exposure to particulate matter was significant at lag 3 days. There were 2177 hospitalizations over the study period, with 201 deaths (9.2%). Premature deaths led to 2035.69 years of life lost. A 10 μg/m^3^ increase in PM_10_ concentrations was correlated with 8.0% of the hospitalizations, which corresponded to 152.67 YLL (81.67 for males and 71.00 for females). The cost generated was approximately US$ 9.1 million in 2016.

**CONCLUSION:**

In this first study conducted in a medium-sized Brazilian city, using the YLL methodology, we identified an excess expense attributable to air pollution.

## INTRODUCTION

In Brazil, in 2016, about US$ 1 billion were spent on hospitalizations due to circulatory system diseases. This comprised over one million hospitalizations with more than 90,000 deaths. In the state of São Paulo, there were more than 250,000 hospitalizations and just over 20,000 deaths, thus generating expenditure of approximately US$ 200 million. In São José dos Campos, a city located in the state of São Paulo, about US$ 3 million were spent on 3,000 hospitalizations, with around 300 deaths.^1^

The factors associated with this morbidity and mortality included physical inactivity, active and passive smoking, hypercholesterolemia and exposure to air pollutants.

Studies have shown positive associations between exposure to air pollutants and presence of cardiovascular diseases. In one study covering the years 2003-2007, the effects of exposure to sulfur dioxide (SO_2_) were found to be significantly associated with mortality due to circulatory diseases, with a relative risk of 1.04 (95% confidence interval, CI: 1.01-1.06).^2^ In another study, an association was found between exposure to environmental pollutants and hospitalizations due to stroke, and it was shown that a 12% increase in the risk of hospitalization (relative risk, RR = 1.137; 95% CI: 1.014-1.276) was associated with a 10 μg/m^3^ increase in the concentration of particulate matter with aerodynamic diameter less than 10 μ (PM_10_).^3^

In Presidente Prudente, a city in the western region of the state of São Paulo that is surrounded by large areas of sugar cane plantations, straw-burning after the harvest is still practiced. This increases the safety of cane cutters, but causes the release of particulate material and gases, in particular NO_2_. These pollutants have been correlated with hospitalizations due to cardiovascular diseases.^4^

An association between exposure to fine particulate matter (PM_2.5_) and hospitalizations due to vascular diseases was identified in another medium-sized city in the state of São Paulo. It was calculated that an excess of 650 hospitalizations, with a cost of US$ 600,000, was caused through an increase in the concentration of this pollutant (PM_2.5_) by 10 μg/m^3^.^5^

A study carried out in Canada using data from 2003 to 2007 also identified a significant association between exposure to nitrogen dioxide (NO_2_) and carbon monoxide (CO) and occurrences of hemorrhagic and ischemic strokes, with relative risks of 1.46 and 1.36. This association showed dose-response behavior with highest risk values in the fourth and fifth quintiles of NO_2_ concentration.^6^

Exposure to air pollution due to particulate matter contributes to cardiovascular morbidity and mortality, such that exposure to PM_2.5_ for a few hours would increase the RR of cardiovascular mortality by approximately 0.4% to 1.0% due to an increase of 10 μg/m^3^ at lag 1 day. Long-term exposure (some years) would increase the relative risk by between 1.06 and 1.76.^7^

In an extensive review on 76 studies published between 2000 and 2018, Bazyar et al.^8^ showed that exposure to air pollutants increased the relative risk or chance (odds ratio, OR) of the need for emergency care in emergency rooms and also of mortality due to cardiovascular diseases such as acute myocardial infarction and hypertension. In another extensive review on 41 studies, higher risk of death due to cardiovascular diseases through exposure to particulate matter was identified.^9^

Positive associations between exposure to pollutants and hospitalizations leading to premature death were identified in a study conducted in Skopje, Republic of North Macedonia, using the Disability Adjusted Life Years (DALY) methodology.^10^ Using this same approach, the economic impact of premature deaths associated with particulate matter concentrations in 29 Brazilian metropolitan regions was evaluated. A total of 20,050 deaths were found, resulting in a cost of US$ 1.7 billion annually.^11^ Also in Brazil, Abe and Miraglia estimated that savings of US$ 15.1 billion per year would be achieved in the city of São Paulo, if the PM_2.5_ concentration were to be reduced in accordance with the standards recommended by the World Health Organization (WHO). This would also prevent about 5,012 premature deaths, i.e. 266,486 years of life lost.^12^

All the studies conducted so far in Brazil have been in major metropolises. Thus, there are no studies in medium-sized cities in Brazil.

## OBJECTIVE

The aim of this study was to estimate the cost of years of life lost (YLL) due to premature deaths in the city of São José dos Campos, Brazil, that were associated with exposure to PM_10_.

## METHODS

### Place of study

São José dos Campos is a city located in the southeastern region of Brazil between the cities of São Paulo and Rio de Janeiro (23° 11’ S and 45° 53’ W). It occupies an area of 1,099 km^2^, has a population of approximately 700,000 inhabitants and has 12 hospitals. This city is an industrial, commercial and service center serving the eastern part of the state of São Paulo and the southern part of the state of Minas Gerais, with a total regional population of approximately two million inhabitants. Some important research centers are installed in this city, such as the National Institute for Space Research (INPE), Technological Institute of Aeronautics (ITA) and São Paulo State University (UNESP). The human development index (HDI) of São José dos Campos is 0.81. The Dutra highway, which is considered to be the most important highway in Brazil, crosses the city and has a flow of approximately 80,000 vehicles per day, including large numbers of heavy vehicles and buses.

### Statistical analysis

This was an ecological time-series study on data relating to hospitalizations due to circulatory system diseases (International Classification of Diseases, 10^th^ edition [ICD-10], chapter X) among residents of São José dos Campos, São Paulo, aged 30 years and over, of both sexes. Information covering the entire year of 2016 was obtained from the DATASUS^13^ website. The data on this website originated from hospital admission authorizations contained in hospital information systems and referred to all municipalities in the state of São Paulo. These data comprise not only accounting information but also information of epidemiological interest such as age, sex, date of admission, diagnosis according to ICD-10 and type of hospital discharge (dead or alive).

The independent variables evaluated in the present study were NO_2_, PM_10_ and O_3_ pollutant concentrations, minimum temperature and relative humidity. These data were obtained from the São Paulo State Environmental Agency (CETESB), which has three monitoring stations in the municipality of São José dos Campos.^14^

The data for the year 2016 were inserted in a spreadsheet for analysis. This analysis used negative binomial regression, instead of Poisson regression, to avoid overdispersion due to possible significant differences between the mean values of hospitalizations and their variance. The model was adjusted for temperature and relative humidity and controlled for day of the week and long-term trend. The analysis used lags of 0 to 5 days between exposure to the pollutant and the outcome.

**Figure 1.**
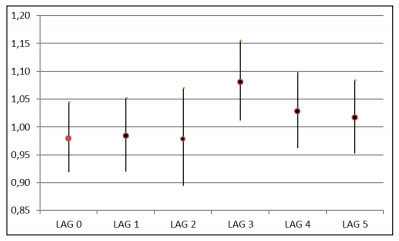
Relative risk with respective 95% confidence intervals, according to a 10 μg/m^3^ increase in particulate matter concentration, from Lag 0 to Lag 5 days.

The negative binomial regression coefficients (βi) with their respective standard deviations were transformed into relative risk (RR) of hospitalization with a confidence interval of 95%, for lags of zero to five days between exposure and outcome, as the effects of this exposure may be evident either on the same day or some days after exposure (lag 0 to lag 5) (Figure 1).

Increases in risk were considered in terms of an increase of 10 μg/m^3^. This was done using the expression (exp (β * DC)), where DC is the increase in concentration of 10 μg/m^3^. Significant values (P-value < 0.05) were identified as the lag that provided the proportional attributable risks (PAR) of the given increase in RR, using the expression PAR = [1-1/RR]. From this, the population attributable fraction (PAF) was estimated using the expression PAF = PAR * N, where N is the number of deaths.

Hospitalizations were included in three groups. Ischemic heart disease (ICD-10 categories I-20 to I-25) (Group 1), congestive heart failure (I-50) (Group 2) and cerebrovascular disease (I-60 to I-64) (Group 3), which corresponded to 70% of all deaths, were selected from among causes in ICD-10 Chapter X. The premature deaths from these diseases were determined, and the financial cost of these premature deaths was calculated using the DALY methodology.

The DALY method is a summary measurement of health expressed by means of a standard indicator in time units (years). It is obtained as the sum of two components: years of life lost (YLL) due to premature death (associated with a specific outcome) in relation to the estimated life expectancy; and years lived with disability (YLD), i.e. the time spent in an unhealthy condition. This method is consistent with the ideals from outcomes such as disease, injury or risk factors, as described by Murray and Lopez.^11^

The YLL component of DALY is obtained through equation 1, as follows:

$$YLL=\frac{{KCe}^{ra}}{{\left(r+\beta \right)}^{2}}\left[e^{-\left(r+\beta \right)\left(L+a\right)}\left[-\left(r+\beta \right)\left(L+a\right)-1\right]-e^{-\left(r+\beta \right)a}\left[-\left(r+\beta \right)a-1\right]\right]+\frac{1-K}{r}(1-e^{-rL})$$ (1)

Where:

r =: discount rate (r = 0.03);
K =: weight-age modulation factor (K = 1);
C =: constant (0.1658);
a =: age at death event;
L =: life expectancy pattern at age a;
β =: weight-age function parameter (β = 0.04).

The variable “a” refers to the age of the individual at the time of death, while the variable L refers to the pattern of life expectancy at age a. These were obtained from the Brazilian Institute for Geography and Statistics (IBGE). For each interval, the value at its onset was considered: for example, for a case within the age range 45-50 years the value was set at 32.2 years for males and 37.0 for females, regardless of age.

These two variables provided the data on the events analyzed that needed to be entered in order to estimate the number of years of life lost due to a given disease. This formula was programmed and developed in an Excel spreadsheet in which the original database was aggregated for calculation. An implementation was introduced in order to make life expectancy comparisons at the age of death considering the same spreadsheet file, thus providing a single output. This output consists of each individual YLL calculation for each event. A value of € 50,000 (euros) was assigned to each individual YLL.

The total number of YLL was obtained by summing the individual YLL results due to pollution-attributable disease mortality, i.e. the part of YLL that was due to the effects of atmospheric pollution within the outcome, using the calculation given in the formula. Thus, YLL due to pollution was obtained via the following formula:

$${YLL}_{pol}=\Sigma \;YLL*PAF$$ (2)

Where:

YLL_pol_ =: YLL attributable to air pollution
Σ YLL =: sum of all individual YLL in the parsed database

The significance level was taken to be alpha = 0.05. This study was conducted using data publicly available from the official source, from which it was not possible to identify the subjects. Therefore, there was no need to submit the study protocol for approval by a research ethics committee.

## RESULTS

This study included 2,177 hospitalizations among individuals aged 30 years and over of both sexes: 1,332 men (61.2%) and 845 women (38.8%). Their diagnoses related to cardiovascular disease groups, as mentioned in the Methods. The daily average number of hospitalizations was 4.8 (standard deviation SD = 2.5), with a range from 0 to 13. There were 201 deaths (9.2% of the hospitalizations): 106 among the males (52.7% of the deaths) and 95 among the females (47.5% of the deaths). Among the males, the proportion of deaths was 7.96%; and among the females, 11.24%. The mortality rate for these three groups of diseases was 29.3 deaths per 100,000 inhabitants.

Table 1 shows the descriptive analysis results from the variables. For the number of hospitalizations, the variance was 11.76, which therefore justified the use of negative binomial regression.

**Table 1.** Descriptive analysis on atmospheric variables, with mean values and their respective standard deviations (SD) and minimum and maximum values (Min-Max), São José dos Campos (SP), 2016

	Mean (SD)	Min-Max
NO_2_^*^ (μg/m^3^)	40.0 (20.4)	4-119
PM_10_^**^ (μg/m^3^)	22.4 (12.4)	4-78
O_3_^***^ (μg/m^3^)	60.8 (21.6)	14-120
Temperature (°C)	27.3 (4.4)	15.8-36.8
Relative humidity (%)	48.5 (13.5)	19-88

^*^Nitrogen dioxide; ^**^particulate matter with aerodynamic diameter less than 10 μ; ^***^ozone.

The modeling coefficients with only one pollutant (unipollutant model) provided by the negative binomial approach are shown in Table 2. The analysis used a multipollutant model and it was only possible to identify significant exposure to PM_10_ at lag 3 days (P-value = 0.021). The relative risk associated with this exposure, in terms of an increase in PM_10_ concentration of 10 μg/m^3^ was RR = 1.081 (95% CI: 1.012-1.155). This represented an excess risk of around 8.1% (Table 3).

**Table 2.** Coefficients and their standard errors, in brackets, provided through analysis on three pollutants separately. São José dos Campos (SP), 2016

Lag^*^	PM_10_	NO_2_	O_3_
Lag 0	0.001162 (0.002339)	0.001875 (0.001295)	0.001378 (0.001688)
Lag 1	0.001796 (0.002310)	0.001831 (0.001283)	0.002084 (0.001661)
Lag 2	0.001613 (0.002321)	0.000535 (0.001299)	-0.000220 (0.001688)
Lag 3	0.004117 (0.002268)	0.000013 (0.001299)	0.000703 (0.001680)
Lag 4	0.000843 (0.002302)	-0.000538 (0.001291)	-0.000099 (0.001664)
Lag 5	0.001432 (0.002280)	-0.000120 (0.001288)	0.000713 (0.001672)

^*^Lag = number of days between exposure and outcome.

**Table 3.** Regression coefficients (Coeff) and respective standard errors (SE) for PM_10_ pollutant (multipollutant model) on all days of the lag structure analyzed, São José dos Campos (SP), 2016.

Lag^*^	Coeff	SE
Lag 0	-0.002080	0.003286
Lag 1	-0.001635	0.003414
Lag 2	-0.002210	0.004573
Lag 3	0.007781^#^	0.003382^#^
Lag 4	0.002765	0.003408
Lag 5	0.001615	0.003290

^*^Lag = number of days between exposure and outcome; ^#^P-value < 0.05.

The results obtained, and the respective percentages, according to the aggregated groups of diseases, are shown in Table 4. It could be seen that the highest percentage of deaths occurred in group 3. On the other hand, there was no significant difference (P-value > 0.05) in YLL values between males and females.

**Table 4.** Multivariate analysis on the variables of type of hospital discharge, mean age, sex and DALY values according to hospitalization groups: ischemic heart disease (Group 1), congestive heart failure (Group 2) and cerebrovascular disease (Group 3). São José dos Campos (SP), 2016

	Group 1	Group 2	Group 3	P-value
**Hospital discharge**				
Alive	1203 (97.6%)	395 (86.8%)	378 (77.1%)	< 0.01
Dead	29 (2.4%)	60 (13.2%)	112 (22.9%)	
**Mean age in years (SE)**	62.3 (10.6)	67.0 (12.1)	64.8 (14.3)	< 0.05
Sex				
Male	801 (65.0)	241 (53.0)	290 (59.2)	< 0.01
Female	431 (35.0)	214 (47.0)	200 (40.8)	
**YLL^#^**				
Male	10.12 (6.75)	9.07 (6.43)	10.04 (5.80)	NS
Female	9.67 (6.29)	8.10 (4.72)	11.48 (7.23)	

^#^mean and standard error (SE); YLL = years of life years; NS = not significant

 The total YLL value was 2035.69 years; the mean value per individual was 9.88 (± 6.18) with values varying between 2.52 and 26.65. The YLL for males was estimated as 1089.02 years (53.5%). According to sex, the average YLL was 9.81 (SD = 6.02) for males and 9.96 (SD = 6.40) for females, with no significant differences.

The YLL values according to sex and diagnosis group are shown in Table 4. The highest YLL value was for group 3 (10.58 ± 6.39) and the lowest was for group 2 (8.54 ± 5.53), but without a significant difference. There were 549.96 YLL within the age range 30 to 50 years (27.02%).

The PAR value for this RR was (1 - 1/1.081) à 0.075. Thus, the resultant PAF value (i.e. the product of total YLL and PAR) was 2035.69*0.075 à 152.67. Out of the total of 2035.69 YLL, 152.67 YLL (81.67 YLL for males and 71.00 for females) were attributed to this increase, with costs of approximately € 7.63 million (approximately US$ 9.16 million) in 2016.

## DISCUSSION

Until now, no studies quantifying the cost of premature deaths associated with exposure to air pollutants had been conducted in a Brazilian medium-sized city, particularly with regard to particulate matter using the YLL DALY component. The costs of these premature deaths were of the order of US$ 9.1 million in the year 2016.

In this study, it was possible to quantify 2035.69 years of life lost, with 1089.02 YLL attributable to males and 946.67 YLL to females. The female response to the risk of death among women represented by the substantial proportion found in this study had already been pointed out in two other studies.^16^^,^^17^

Studies conducted in Brazil have already shown the costs of hospitalizations associated with exposure to all air pollutants. With a reduction in pollutant concentrations, there is a reduction in the costs of these hospitalizations to the National Health System (Sistema Único de Saúde, SUS). It was found in São José do Rio Preto, state of São Paulo, that a reduction in the PM_2.5_ concentration would lead to avoidance of around 600 hospitalizations, with cost savings of around US$ 550,000.^6^ This was also demonstrated in studies conducted in Cuiabá, state of Mato Grosso, with savings of about US$ 30,000 regarding hospitalizations among children^15^ and around US$ 100,000 regarding hospitalizations among the elderly^16^ due to respiratory diseases. For cardiovascular diseases, it was possible to estimate a reduction of US$ 150,000 in Taubaté.^17^ Regarding the reduction in PM_2.5_ concentrations, there would be a reduction of 256 hospitalizations and cost savings of approximately US$ 60,000 in São José do Rio Preto.^18^

A study carried out in Skopje, Republic of North Macedonia, on hospital admissions due to respiratory diseases in 2012, identified around 1200 premature deaths, costing between € 570 million and €1470 million, when the PM_2.5_ concentration was 49.2 μg/m^3^. Lowering PM_2.5_ levels to the European Union recommended limit of 25 μg/m^3^ would decrease these deaths by 45%.^7^ The PM_2.5_ concentration in Skopje corresponded to around 80 μg/m^3^ in PM_10_ values, i.e. much higher than the values recorded in São José dos Campos. The YLL values were obtained using the AirQ+ software, which quantifies the health impact of exposure to air pollutants and was developed by WHO.

A study was conducted in 29 Brazilian metropolitan regions using the DALY methodology, to assess the economic impact of premature deaths associated with air pollution. A total of 20,050 deaths were considered to be due to exposure to particulate matter, and these generated a cost of US$ 1.7 billion annually.^11^ In that study, deaths from all causes that occurred among subjects of both sexes aged 30 years or over were considered, and it was determined that these deaths corresponded to approximately 250,000 YLL. This was 100 times greater than what we calculated in our study, but it is difficult to compare the data because all deaths were included in that study, whereas we included only a few diagnoses.

In São Paulo, Brazil, the health impact associated with air pollution was assessed for the years 2009 to 2011. It was determined that reducing the concentrations of particulate matter and ozone would prevent more than 5,000 premature deaths, which corresponded to a gain of 266,486 life years, with savings of US$ 15.1 billion per year. For a reduction in the average PM_2.5_ level in São Paulo of 5 μg/m^3^, about 1,724 deaths would be prevented and there would be savings of US$ 4.96 billion.^12^ It is also difficult to make comparisons with the results from that study, since the diagnoses of cardiovascular diseases were different, in addition to differences in study duration (three years for that study) and in the population studied. Nonetheless, the results from that study also showed that reductions in particulate matter concentrations would lead to decreases in the number of deaths, with an important reduction in costs for the healthcare system.

In Kuwait, where PM_10_ concentrations are of the order of 170 ­μg/­m³, the AirQ+ software was used to show that reducing these concentrations could increase life expectancy in the group of subjects aged 30 years or over.^19^

According to Gao et al., in a review of the DALY method, it could be seen from the existing studies that use of DALY was advantageous in relation to conventional environmental impact assessments for quantifying and comparing the risks from environmental pollution. However, they concluded that further studies were still needed in order to standardize the assessment methods relating to the effect on health of various pollutants under various circumstances, prior to calculating DALY.^20^

In Guangzhou City, China, between 2004 and 2007, the YLL through cardiovascular mortality due to exposure to air pollution was estimated. It was calculated that a 10 μg/m^3^ increase in NO_2_, SO_2_ and PM_10_ would resulting in daily average YLL corresponding to 248, 87.5 and 73.7 for deaths from cardiovascular disease (CVD), stroke and ischemic heart disease (IHD) respectively. The effects of air pollutants on YLL were immediate and lasted for two days.^21^

This study may have some limitations. Among these, it was developed using secondary data: even though these data came from an official source (DATASUS), they may have contained misdiagnoses, since the main purpose of DATASUS is to record financial information. In addition, the hospitalization data referred only to occurrences within the public system through SUS, thus excluding private hospitalizations or occurrences though health plans or health insurance operators. Furthermore, factors such as passive smoking, dietary habits and lifestyle were not considered because they are not available through DATASUS. The time period used in this study was one year only.

## CONCLUSIONS

Despite these possible limitations, this study not only presented unpublished data from a medium-sized city in Brazil but also quantified the cost of premature deaths due to some cardiovascular diseases. If these deaths were avoided, the cost savings could be allocated to other healthcare needs in the city. In addition, it was possible to estimate the importance of YLL in an important economically active age group.
